# Corneal Stroma Regeneration with Acellular Corneal Stroma Sheets and Keratocytes in a Rabbit Model

**DOI:** 10.1371/journal.pone.0132705

**Published:** 2015-07-13

**Authors:** Xiao Yun Ma, Yun Zhang, Dan Zhu, Yang Lu, Guangdong Zhou, Wei Liu, Yilin Cao, Wen Jie Zhang

**Affiliations:** 1 Department of Plastic and Reconstructive Surgery, Shanghai 9th People’s Hospital, Shanghai Jiao Tong University School of Medicine, Shanghai Key Laboratory of Tissue Engineering, National Tissue Engineering Center of China, Shanghai, China; 2 Department of Ophthalmology, Shanghai Guanghua Integrative Medicine Hospital, Shanghai, China; Cedars-Sinai Medical Center; UCLA School of Medicine, UNITED STATES

## Abstract

Acellular corneal stroma matrix has been used for corneal stroma engineering. However, because of its compact tissue structure, regrowth of keratocytes into the scaffold is difficult. Previously, we developed a sandwich model for cartilage engineering using acellular cartilage sheets. In the present study, we tested this model for corneal stroma regeneration using acellular porcine corneal stroma (APCS) sheets and keratocytes. Porcine corneas were decellularized by NaCl treatment, and the APCS was cut into 20-μm-thick sheets. A rabbit corneal stroma defect model was created by lamellar keratoplasty and repaired by transplantation of five pieces of APCS sheets with keratocytes. Six months after transplantation, transparent corneas were present in the experimental group, which were confirmed by anterior segment optical coherence tomography examination and transmittance examination. The biomechanical properties in the experimental group were similar to those of normal cornea. Histological analyses showed an even distribution of keratocytes and well-oriented matrix in the stroma layer in the experimental group. Together, these results demonstrated that the sandwich model using acellular corneal stroma sheets and keratocytes could be potentially useful for corneal stroma regeneration.

## Introduction

Corneal dysfunction due to injury or infection often leads to vision impairment and a poor quality of life, and is the third leading cause of blindness worldwide [1, 2]. The usual treatment for corneal blindness is keratoplasty [3]. In classic penetrating keratoplasty, the whole cornea, including the epithelial layer, stromal layer, and endothelial layer, is replaced. Recently, lamellar keratoplasty, in which only the diseased layer is replaced without removing the whole cornea, has been developed [4, 5]. However, the poor availability of donor corneas restricts the wide application of this technique [6–8]. Several attempts have been made to regenerate human corneas using a tissue engineering approach. The corneal epithelial layer, stromal layer, and endothelial layer have been regenerated [9, 10], and significant progress has recently been made towards clinical use of these biomaterials [11–14].

In some corneal dysfunctions, such as stromal keratitis and keratoleukoma, only stromal layer regeneration is required [15]. Tissue engineering of the cornea stromal layer presents particular challenges because of its specialized functions both in transmitting and focusing light, as well as in resisting intraocular pressure [9]. The selection of a scaffold that provides a three-dimensional structure for the growth of cells and maintenance of their functions is crucial to meet these functional requirements. Biodegradable materials, such as polylactic acid, polyglycolic acid, collagens, and acellular corneal stroma matrix, have been used for corneal stroma engineering [9, 16–19]. Among these, the acellular corneal stroma matrix, which contains the natural components and structure of corneal stroma, has been given special emphasis in corneal engineering [20]. However, because of its compact tissue structure, it is difficult to remove the cellular components [21]. In addition, regrowth of the scaffold with keratocytes is problematic. When acellular porcine corneas were implanted into rabbit corneas, no immune reactions occurred, and the turbid corneas became clear. However, few keratocytes were observed in the graft, even at 12 months after transplantation [22]. Without the growth of keratocytes into the matrix, the long-term outcomes of using this scaffold for corneal stroma regeneration are uncertain. Many attempts have been made to overcome this problem, but with limited success [14, 23, 24].

A similar difficulty has also existed in other acellular matrices with compact tissue structures, such as dermis and cartilage [25, 26]. Previously, we developed an efficient sandwich model for cartilage engineering using acellular cartilage sheets [27]. Briefly, cartilage was cut into 10-μm-thick sheets and decellularized to obtain acellular cartilage sheets. Chondrocytes were then seeded on the sheets and stacked layer by layer to obtain a cell-sheet sandwich. After several weeks of *in vitro* or *in vivo* incubation, a native-like cartilage with predesigned shape was produced [27]. Based on these results, we hypothesized that a sandwich model might also be applicable to corneal stroma engineering. In the present study, we initially obtained acellular porcine corneal stroma sheets, then combined them with keratocytes to test the corneal stroma regeneration sandwich model in a rabbit model.

## Materials and Methods

### Animals

Whole porcine eyes were collected from Yorkshire swine (6 months old, weighing 105–125 kg) within 3 h postmortem by an overdose of urethane. The eyes had an integral corneal surface with a horizontal corneal diameter of 12–14 mm. Young adult New Zealand white rabbits of either gender, 10 weeks of age, weighing 2–3 kg, were used as recipients. All animal experimental protocols were approved by the Ethics Committee of Shanghai Jiaotong University School of Medicine, Shanghai, China.

### Preparation of acellular porcine corneal stroma (APCS) sheets

Porcine cornea with a 2-mm scleral ring was removed with a 16-mm corneal trephine. The epithelium and endothelium were removed by enzymatic digestion with a solution of 4 mg/mL of Dispase II (Roche, Mannheim, Germany) for 45 min at 37°C. After a 10-min wash with 10% antibiotic and antimycotic solution (Invitrogen-Gibco, Carlsbad, CA, USA) in phosphate-buffered saline (PBS), NaCl-based decellularization of whole corneal stroma was performed as previously described [14]. Briefly, the corneal stroma was immersed in 1.5 M NaCl with continuous shaking (200 rpm) for 12 h at room temperature, followed by three washes with PBS for 30 min at room temperature with continuous shaking. The decellularized stroma was then introduced between several layers of filter paper (Whatman 3MM; Qiagen, Valencia, CA, USA) to eliminate the liquid. They were sequentially dehydrated in 10% sucrose for 1 h, 20% sucrose for 1 h, and 30% sucrose overnight at 4°C. After embedding in O.C.T. Compound (Leica, Wolfurt, Germany), APCS sheets of 20 μm were produced by freeze-sectioning. The sheets were washed with distilled water, air dried at room temperature, and sterilized by irradiation with ultraviolet light overnight. For histological analysis, the sheets were stained with hematoxylin and eosin. Cell nuclei were identified by staining sheets with 4',6-diamidino-2-phenylindole (DAPI; Biomol, Plymouth Meeting, PA, USA). Masson staining was performed to detect distribution of collagen.

### Cell culture

Corneal keratocytes (cultured stromal cells) were prepared according to the procedure of Beales et al. [28], with some modifications. After removing the epithelium and endothelium from the cornea, the remaining stroma was treated with 0.25% trypsin in Hank’s Balanced Salt Solution (HBSS) (Invitrogen, Gaithersburg, MD, USA) for 4 h at room temperature, and washed with HBSS. The stroma was then digested with a solution of 1 mg/mL collagenase in HBSS for 10 min to remove remaining epithelial and endothelial cells. Finally, the stroma was digested with 3 mg/mL collagenase in Dulbecco’s Minimum Essential Medium (DMEM) at 37°C for 4 h. Cells were resuspended in 10 mL DMEM containing 1% fetal bovine serum, plated on 60-mm dishes (6–8 × 10^5^ cells/dish), and allowed to attach overnight. The medium was replaced by DMEM without serum the next day. The medium was changed every 2–3 days. Cells at passage 2 and 3 were used in the experiments.

### Scanning electron microscopy (SEM) and transmission electron microscopy (TEM)

For SEM, APCS sheets seeded with keratocytes were rinsed with PBS and fixed overnight in 0.05% glutaraldehyde at 4°C. After dehydration through a graded series of ethanol, samples were critically point dried and examined using SEM (JEOL-6380LV, JEOL, Japan). To analyze collagen fiber arrangement, samples were fixed in 2.5% glutaraldehyde, embedded in resin, and cut into ultrathin sections. The sections were analyzed by TEM (H600; Hitachi, Japan).

### Surgical procedures

**Fig 1:**
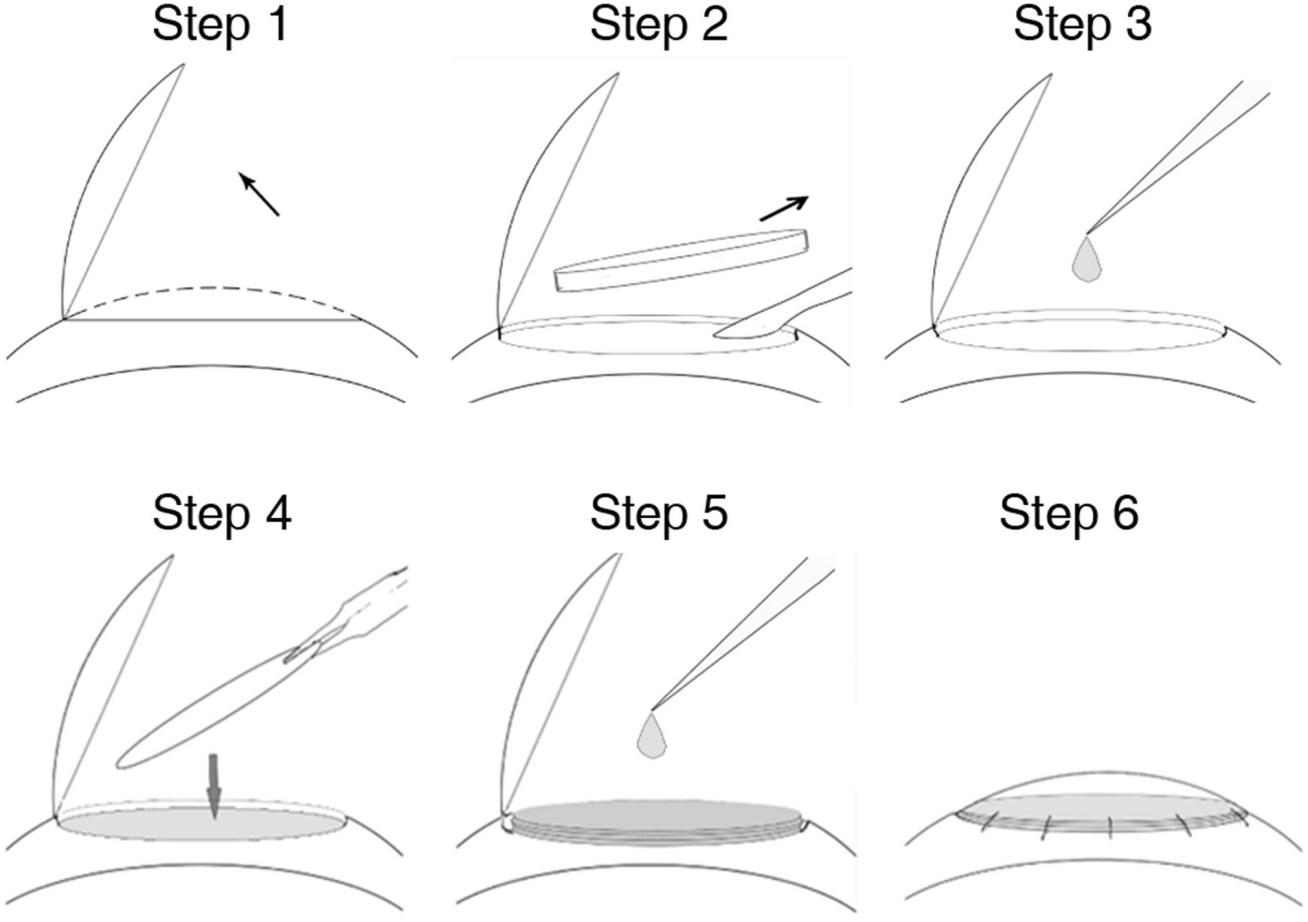
Surgical procedures involving the creation of corneal stromal defects by lamellar keratoplasty (LKP), followed by repair using transplantation of acellular porcine corneal stroma (APCS) sheets with keratocytes.

Stroma defects were created by lamellar keratoplasty (LKP) and repaired by APCS sheets and keratocytes. LKP was performed according to previous reports [23, 28]. A diagram of the surgical procedure is shown in Fig 1. Briefly, animals were anesthetized with an intravenous injection of a mixture of 20 mg/mL ketamine and 2 mg/mL xylazine diluted in saline. A 100-μm-deep, 8-mm circular incision was made on the rabbit cornea using a Barraquer trephine. A stroma flap was then made using an operating knife along a natural uniform stratum in the corneal stroma (step 1). Then a 100-μm-thick stroma layer was removed (step 2). The operated eyes were then divided into four groups: the defect-only group (n = 6), the thick APCS group (n = 6), the APCS sheets group (n = 6), and the APCS sheets + cell group (n = 6). In the APCS sheets + cell group, 5 μl of keratocytes (1 × 10^7^/mL) were seeded into the defective area (step 3), followed by placing a piece of APCS sheet (20 μm thick, step 4). Another drop of cells was seeded, followed by stacking another APCS sheet on top of the first sheet (step 5). The procedure was repeated until five sheets were stacked. The stromal flap was then flipped over and sutured using five interrupted 10–0 nylon sutures (step 6). In the defect-only group, the stromal flap was flipped over and sutured without implanting any cells or scaffold. In the thick APCS group, a thick APCS sheet (100 μm) was implanted with 25 μL keratocytes (1 × 10^7^/mL). In the APCS sheet group, five pieces of APCS sheets (20 μm/sheet) were implanted with 25 μL PBS. For all groups, tobramycin and dexamethasone eye drops were used daily for 7 days after LKP. Clinical examinations were followed up for 6 months, which included inspecting corneal optical clarity, plus characterization of neovascularization and inflammation. A previously described scoring system was used to measure the degree of corneal opacity [29]. Contralateral corneas served as normal controls (n = 6).

### Anterior segment optical coherence tomography (AS-OCT) examination

AS-OCT examination was performed postoperatively at 6 months. All subjects underwent imaging with spectral-domain OCT (Retina Scan 3000; NIDEK, Japan), using an anterior segment probe. The rabbits were anesthetized and an artificial tear was applied to prevent corneal drying. The rabbits were positioned and stabilized on the chin rest, and the probe was adjusted, with centering as much as possible in the corneal vertex and the center of the pupil. Images with high resolution from 50 scans were automatically combined into a single process. The implanted scaffolds, scars, and gaps could be identified by different intensities on the image.

### Ultrasonic pachymeter analysis

Central corneal thickness measurements were performed postoperatively at 6 months using an ultrasonic pachymeter (SW-2000, Tianjin Suowei Electronic Technology, Tianjin, China). Under topical anesthesia, a solid contact ultrasonic probe was placed on the cornea center, and the corneal thickness was calculated as the width of the first peak in the oscilloscope signal, which represented the distance between the air-cornea and cornea-anterior chamber boundaries. The thickness was determined by an average of 10 measurements from each eye.

### Light transmittance assay and biomechanical testing

Rabbits were sacrificed at 6 months post-operation by an overdose of urethane. Corneas were harvested, fixed to cuvettes, and the light transmittance was recorded at 400–700 nm using a UV/Vis/NIR spectrometer (Lambda 9, Perkin Elmer, Waltham, MA, USA). For biomechanical testing, fresh corneas were measured using a biomechanical analyzer (Instron 4411, Canton, MA, USA). Corneas measuring 8 × 4 mm^2^ were fixed on two grippers and drawn along the longitudinal direction at a constant speed of 10 mm/min until complete rupture occurred to acquire the maximal load (Newton). Young’s modulus (MPa) was calculated from the linear slope of the stress-strain curve. All data were automatically processed by Instron analysis computer software (Instron 4411).

### Histological examination

Specimens were fixed in 4% paraformaldehyde at room temperature for 24 h, dehydrated stepwise using ethanol, immersed in xylene, and embedded in paraffin. Paraffin sections were cut 4 μm thick, deparaffinized, and stained with Mayer’s hematoxylin and eosin.

### Statistical analysis

Data were expressed as the mean ± standard deviation. Comparisons between groups were analyzed by Student’s *t*-test or ANOVA for experiments with more than two subgroups. A value of *P* < 0.05 was considered statistically significant.

## Results

### Characterization of APCS

**Fig 2:**
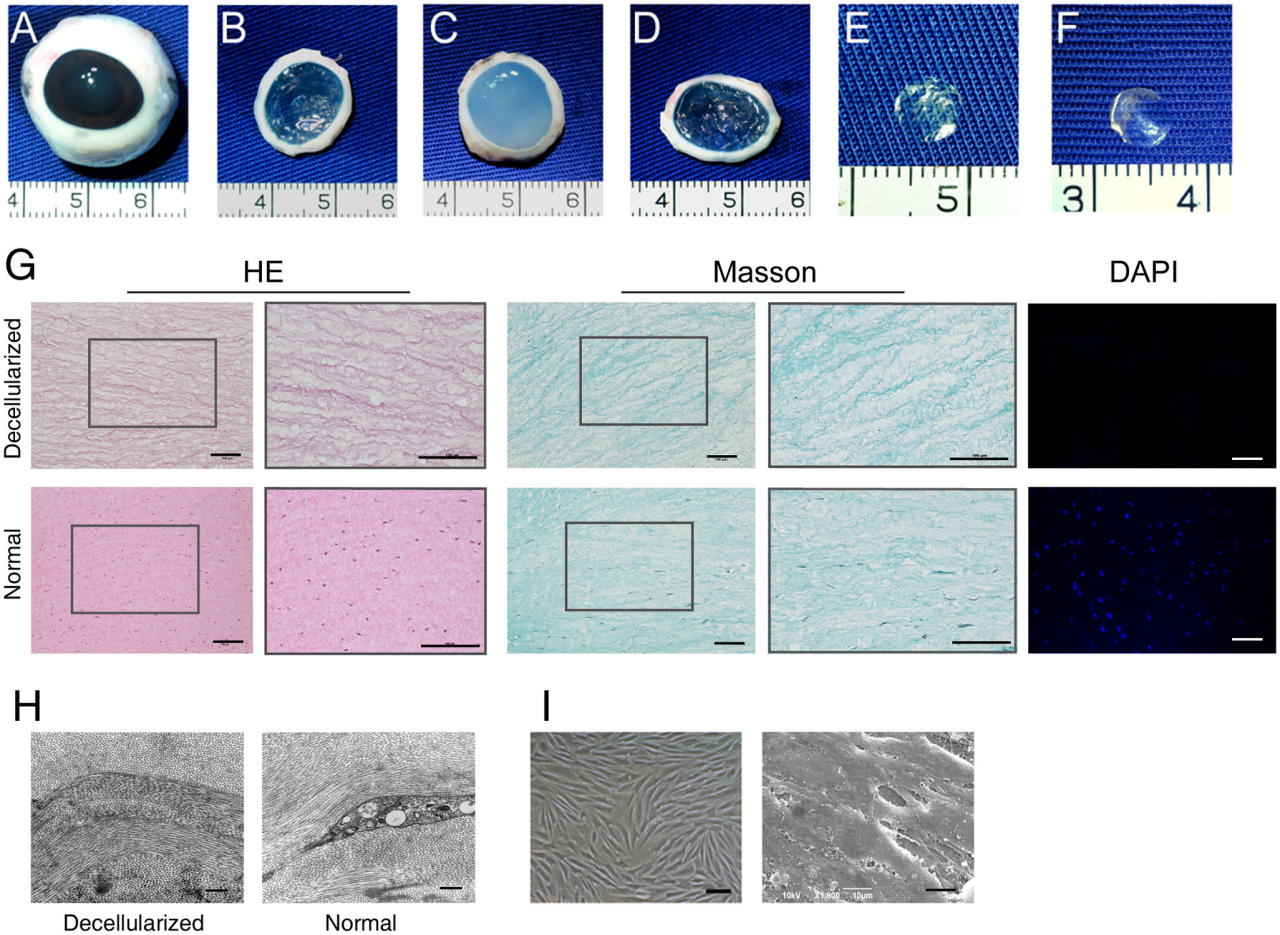
Characterization of the APCS. (A) Porcine eye, (B) cornea with 2-mm scleral ring, (C) after the NaCl decellularization process, (D) after dehydration with sucrose, (E) a 20-μm-thick APCS, and (F) a 100-μm-thick APCS. (G) Histology of the APCS and normal corneal stroma using hematoxylin and eosin staining, Masson staining, and 4',6-diamidino-2-phenylindole (DAPI) staining. Scale bars: 100 μm. (H) Ultrastructure of APCS and normal cornea stroma by transmission electron microscope. Scale bars: 0.5 μm. (I) Keratocytes in a tissue culture plate (left, scale bar: 100 μm) and keratocytes grown on the APCS (right, scale bar: 10 μm).

Porcine eyes were collected within 3 h postmortem (Fig 2A). The corneas were removed with a 2-mm scleral ring (Fig 2B). After enzymatic digestion and NaCl decellularization processes, the APCS was obtained (Fig 2C). The transparency of the APCS increased after dehydration with sucrose (Fig 2D). The APCS was then frozen and cut into pieces with thicknesses of 20 μm and 100 μm. After several washes and air drying at room temperature, the transparent APCS scaffolds were ready for use (Fig 2E and 2F).

Complete removal of the epithelial, endothelial, and stromal cells was confirmed by histological analyses of the APCS after hematoxylin and eosin staining (Fig 2G). The removal of cells was confirmed by DAPI staining of the cell nucleus (Fig 2G). Masson staining showed that the collagen fibrils were slightly loosened after decellularization (Fig 2G). Ultrastructure observed by TEM showed that the collagen fibril orientation was well preserved, with no cells observed in the APCS (Fig 2H). The biocompatibility of the APCS was tested by seeding keratocytes on the APCS sheets. After 7 days of incubation, SEM analysis showed that cells attached on the sheet and secreted matrix (Fig 2I).

### Transplantation of APCS in a rabbit LKP model

**Fig 3:**
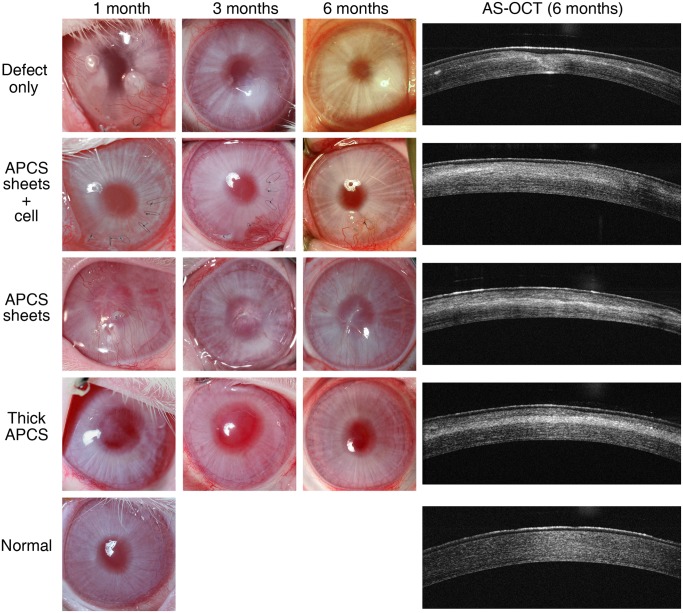
Gross views of repaired cornea postoperatively at 1, 3, and 6 months, and anterior segment optical coherence tomography (AS-OCT) examination postoperatively at 6 months.

A rabbit corneal stroma defect model was created by LKP and repaired by transplantation of APCS with or without keratocytes. The gross views of each cornea were photographed postoperatively at 1, 3, and 6 months. As shown in Fig 3, 6 months after transplantation, transparent corneas were present in the APCS sheets + cell group and thick APCS group. However, severe corneal opacity was observed in the defect-only group without treatment and the APCS sheets group without seeding cells. The detail of the corneal opacity scored at 6 months post-operation in each animal is listed in Table 1. AS-OCT examination postoperatively at 6 months showed an even signal from the stromal layer in the APCS sheets + cell group, which was similar to that of normal cornea (Fig 3). However, irregular signals from the stromal layer were observed in the defect-only group and the APCS sheets group, which were consistent with the gross observations. In the thick APCS group, a layer of high signal intensity with clear boundaries in the stroma was observed, which likely resulted from the undegraded APCS scaffold.

**Table 1:** Corneal opacity scored at 6 months post-operation.

Animal No. in each group	Defect-only (group 1)	APCS sheets + cell (group 2)	APCS sheets (group 3)	Thick APCS (group 4)
1	++	+	++	+
2	+	-	++	+
3	++	-	+	-
4	+++	-	++	+
5	++	+	++	++
6	++	+	++	+

- clear and compact cornea;

+ minimal opacity;

++ mild deep (stromal) opacity with pupil margin and iris vessels visible;

+++ moderate stromal opacity with only pupil margin visible.

Mann-Whitney U analyses: group 2 was very significantly different from group 1 (Z = -2.760, p = 0.004); group 3 was not significantly different from group 1 (Z = -0.527, p = 0.699); group 4 was significantly different from group 1 (Z = -2.245, p = 0.041).

### Corneal thickness, transmittance, and biomechanical properties

**Fig 4:**
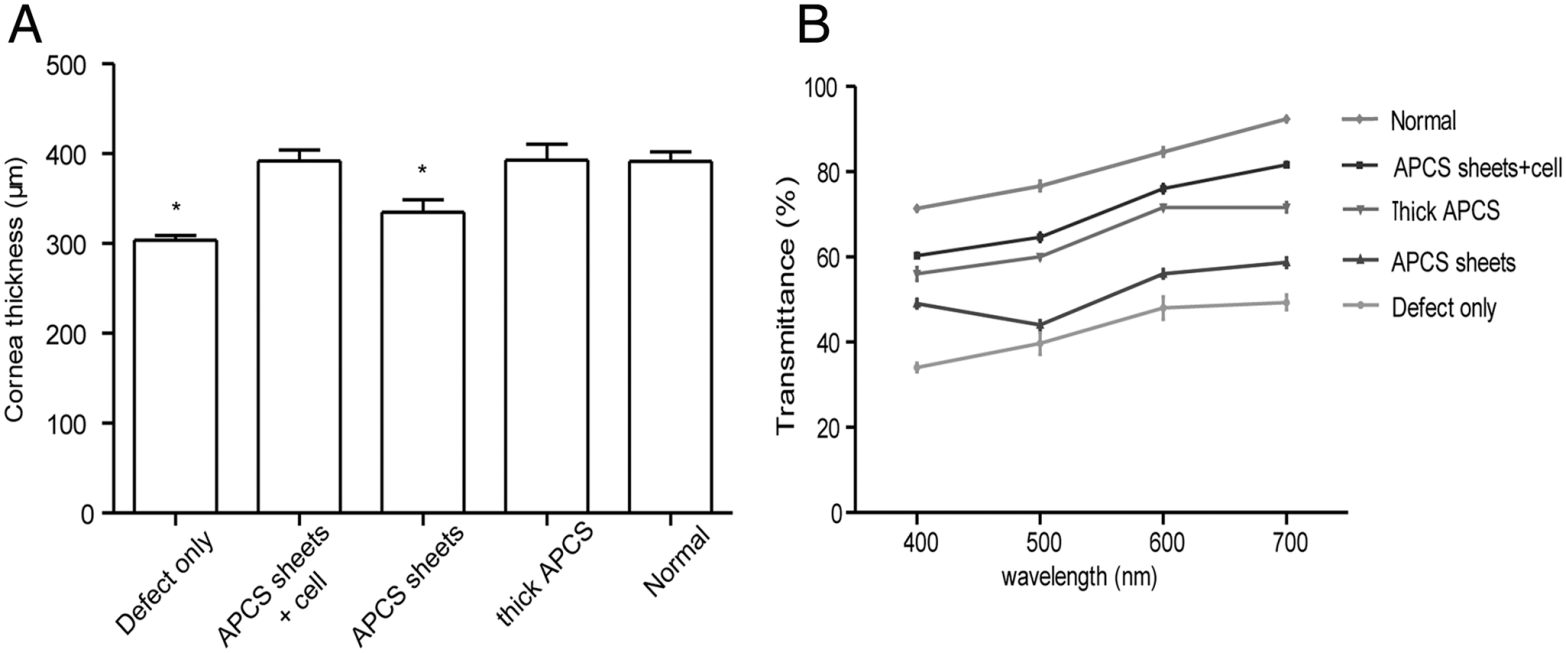
The thickness and transmittance of repaired cornea postoperatively at 6 months. (A) The thickness of each cornea was measured using an ultrasonic pachymeter (n = 6 in each group). (B) The light transmittance of each cornea was recorded using a UV/Vis/NIR spectrometer (n = 6 in each group). *P < 0.05 compared with normal cornea.

The thickness of each cornea postoperatively at 6 months was measured using an ultrasonic pachymeter. The thicknesses of corneas in both the defect-only group and APCS sheets group were significantly thinner than that of normal cornea, while the thicknesses of corneas in the APCS sheets + cell group and thick APCS group were similar to normal cornea (Fig 4A). Corneal transmittance examination showed that at all wavelengths of light tested, similar transmittances as normal cornea were observed in the APCS sheets + cell group and thick APCS group, while lower light transmittances were observed in both the defect-only group and APCS sheets group (Fig 4B).

**Fig 5:**
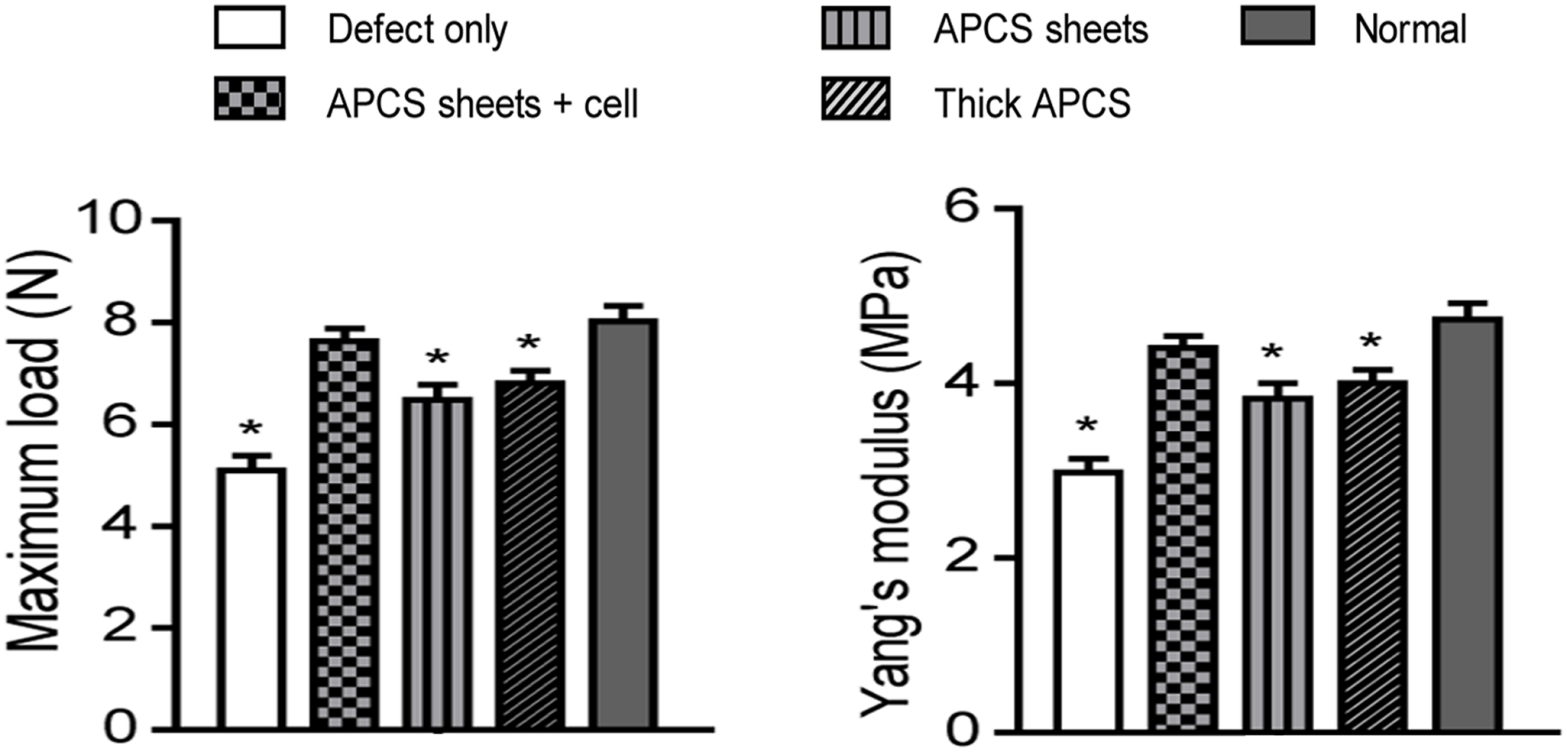
Biomechanical properties of repaired cornea postoperatively at 6 months (n = 6 in each group). *P < 0.05 compared with normal cornea.

To further evaluate the strength of the repaired stroma, the biomechanical properties of each cornea were measured. As shown in Fig 5, the biomechanical properties, including maximum loading and Young’s modulus, in the APCS sheets + cell group were similar to those of normal cornea, while poorer biomechanical properties were observed in the other three groups.

### Histological analyses

**Fig 6:**
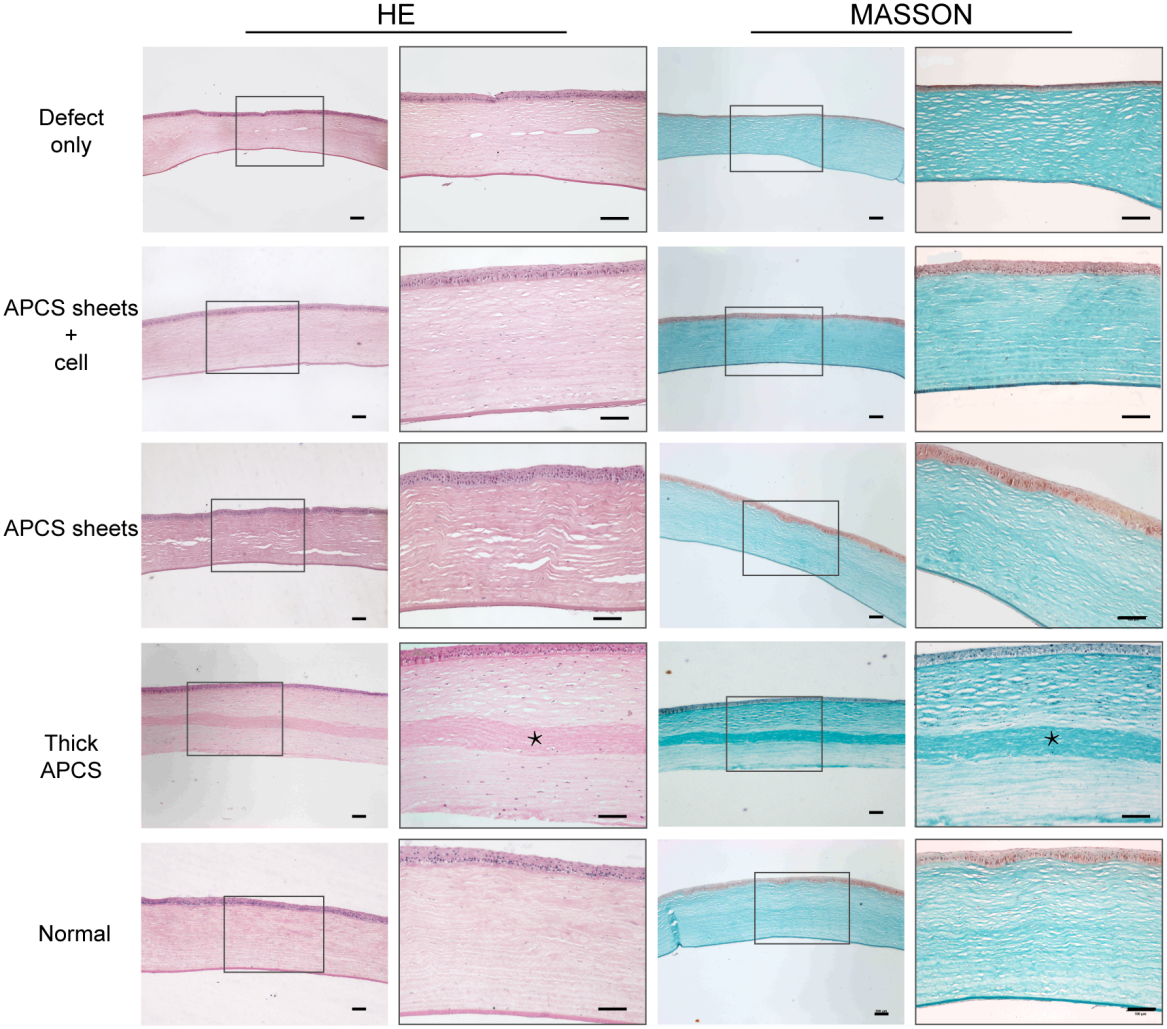
Histological analysis of repaired cornea using hematoxylin and eosin staining and Masson staining. *Undegraded APCS. Scale bars in low-magnification photographs: 500 μm. Scale bars in high-magnification photographs: 100 μm.

To confirm the regeneration of corneal stroma, all cornea samples were collected postoperatively at 6 months, and histological examination was performed after hematoxylin and eosin staining and Masson staining. As shown in Fig 6, in the thick APCS transplanted group, a clear scaffold with darker Masson staining was observed in the stroma, but few cells were observed in the scaffold. However, the scaffold was barely observed in the APCS sheets group and the APCS sheets + cell group. The histology in the APCS sheets + cell group was almost identical to normal cornea, with an even distribution of keratocytes in the stroma, while a thinner cornea with a slightly looser stroma was observed in the APCS sheets group compared with normal cornea. Consistent with the ultrasonic pachymeter measurements, a thinner cornea was observed in the defect-only group.

## Discussion

Scaffolds, which provide a three-dimensional structure for the growth of cells, play a crucial role in tissue engineering. Theoretically, acellular matrix derived from the same type of tissue that contains the native components and maintains the native tissue structure would be expected to be the ideal scaffold for targeted tissue regeneration. Acellular corneal stroma matrix has been used for corneal engineering [14, 18, 20, 23]; however, because of the low porosity of the matrix, cells had difficulty migrating into the scaffolds to achieve a natural cornea [22]. Based on our experience using acellular cartilage sheets for cartilage engineering, we chose the sandwich model using APCS sheets for corneal stroma regeneration. By loading APCS sheets and keratocytes layer by layer into the corneal stroma defects, a transparent cornea was achieved after 6 months. Histological analysis showed that keratocytes distributed evenly in the regenerated stroma, indicating that the regenerated stroma was very similar to normal cornea.

Acellular scaffolds have been widely used in tissue regeneration. A key step in preparing such scaffolds is to remove, as much as possible, the cell components while maintaining their biological activities and histological structure. Efficiently removing cell components by intensive chemical or physical treatments while maintaining the biological activities and histological structure has been difficult [21]. The latter parameters are particularly important for acellular corneal stroma because the transparency of the cornea relies on the well-organized structure of collagen fibers in the matrix [9, 30]. Many attempts, including laser and lyophilization treatments, have been made to improve the porosity of the acellular scaffolds [31–33]. Using a gentle NaCl solution treatment, as previously reported by others [14], we could efficiently remove the keratocytes (Fig 2G). Using this approach, the histological structure and well-organized collagen fibers were preserved (Fig 2G), and the biocompatibility of the acellular sheets was sufficient to support the growth of keratocytes (Fig 2H). Furthermore, by cutting the APCS into pieces and seeding keratocytes layer by layer, we could achieve a more even distribution of cells in the grafts (Fig 6). Thus, this methodology could overcome the problems associated with the low porosity of the scaffold, while taking full advantage of the acellular matrix for new tissue formation.

Similar to previous observations from Hashimoto et al. [22], implantation of thick APCS (100 μm) could achieve a transparent cornea 6 months after the operation. However, very few cells were observed in the scaffolds even though the same amount of keratocytes were seeded during the operation (Fig 6). The implanted scaffolds could be clearly observed by AS-OCT measurements and histological analyses (Figs 3 and 6). At this time point, the corneal thickness of this group was similar to that of the normal cornea (Fig 4A), indicating that degradation of the scaffold was slow. However, in the APCS sheets group, involving five pieces of thin sheets (20 μm/sheet, same thickness in total as the thick APCS group) implanted without seeding cells, the thickness of cornea postoperatively at 6 months was close to that of the defect-only group, and thinner than the normal cornea. No clear APCS sheets were observed by OCT measurements and histological analyses (Figs 3 and 6), indicating that most of the scaffold had been degraded. Clearly, without seeding cells, the native keratocytes could not efficiently migrate into the sheets, even though there were spaces between sheets. However, creating pores on each APCS sheet might be helpful for cell infiltration, and should be investigated in future studies.

Another important finding from the APCS sheets group suggested that the APCS would be completely degraded eventually. In addition, the absorbed matrix likely could not be regenerated by native keratocytes. Therefore, the long-term outcome of transplanting with thick APCS may not be successful [22]. Compared with the results from the APCS sheets group and the APCS sheets + cell group, seeded keratocytes played an important role in the stromal regeneration process. Since the cells haven’t been labeled before transplantation, it is difficult to tell the fate of seeded cells at 6 months post-operation. No obvious stroma regeneration was observed in the APCS sheets group (Fig 6), suggesting that the native keratocytes could not efficiently migrate into the sheets. Thus, stromal cells in the repaired area of the APCS sheets + cell group are likely from survived seeded-cells rather than from migrated host cells. Further studies of using labeled cells for transplantation would address this question. When considering the possible future clinical application of this approach, autologous keratocytes are difficult to obtain. Thus, alternative cell sources should be tested. In our previous study, we reported that dermal fibroblasts could switch their phenotype to keratocytes in the cornea stromal environment [34]. Using such cells should therefore be investigated in the future.

Besides the acellular corneal stroma matrices, many biomimetic scaffolds, such as silk fibroin-chitosan [35], self-assembling peptides [36], and recombinant human collagen [37], have been successfully used in corneal stroma regeneration in animal models as well as in clinical trails. These scaffolds possess good biocompatibility and immune compatibility. However, the mechanic properties of these scaffolds are relatively low that they might be only suited for injection [36, 37]. The APSC sheets are strong enough to be stacked and maintain their 3-demensional structure in culture (unpublished data). Engineering a whole cornea with epithelial, stromal, and endothelial layers using APSC sheets is under investigation.

## Conclusions

We have described a sandwich model using acellular corneal stroma sheets and keratocytes for corneal stroma regeneration. This approach could be a potential clinical method for repairing corneal stromal defects. However, using thick acellular corneal stroma matrices is not advisable until a long-term follow-up study is completed.
